# Mandibular Advancement Device (MAD) for Obstructive Sleep Apnea in an Edentulous Patient

**DOI:** 10.7759/cureus.32896

**Published:** 2022-12-24

**Authors:** Ayesha Burugpalli, Pallavi Chikhale, Ankit Galav, Deepanshu Sharma, Shivani Puranik, Kapil Paiwal

**Affiliations:** 1 Department of Prosthodontics, Daswani Dental College & Research Center, Kota, IND; 2 Department of Oral & Maxillofacial Pathology, Daswani Dental College & Research Center, Kota, IND

**Keywords:** splint fabrication, oropharyngeal volume, edentulous patients, obstructive sleep apnea (osa), mandibular advancement device

## Abstract

Mandibular advancement devices have been efficiently used for the treatment of mild to moderate obstructive sleep apnea (OSA) by forward positioning of the tongue-mandible complex with a resultant increase in oropharyngeal volume. However, the literature on the treatment of edentulous patients is limited. This clinical report describes a clinical and laboratory method for the fabrication of a mandibular advancement device in a 75-year-old completely edentulous patient with mild OSA.

## Introduction

Obstructive sleep apnea syndrome (OSAS) is a critical disorder with repetitive cessation of respiration owing to narrowing or collapse of the upper airway during sleep [1,2]. Repetitive episodes of apnea (complete cessation of airflow), hypopnea (partial reduction in airflow), oxygen desaturation, and sleep fragmentation are the common symptoms experienced by these patients [3]. Around 14-30% of male adults and 5-15% of female adults are affected by this medical problem [4,5]. In a study conducted in the Indian population, about 13.7% of the participants were diagnosed with OSA [6].

Patients suffering from OSAS have an inadequate oxygen supply due to apnea, which increases the blood carbon dioxide pressure and leads to the eventually awakening of the patient momentarily, to breathe in, and return to sleep without consciously remembering the episode [3,4]. Along with excessive daytime sleepiness, patients with OSA are known to manifest impaired neuropsychological functioning, mood disturbances, and a decrease in quality of life relative to healthy controls [7,8].

The severity of OSA is measured as the apnea-hypopnea index (AHI), i.e., the number of apneas per hour of sleep [8]. According to the American Academy of Sleep Medicine (AASM), it is categorized into mild (5-15 events/hour), moderate (15-30 events/hour), and severe (>30 events/hour).

Current guidelines put forward non-surgical treatment options involving continuous positive airway pressure (CPAP), lifestyle changes, and position therapy as first-line treatments. Second in line are oral appliances and surgical approaches (uvulopalatopharyngoplasty and tracheostomy) based upon the etiology and degree of severity [1]. Over the past two decades, CPAP therapy has been considered the most beneficial treatment for OSA [8-10].

In mild and moderate OSA patients mandibular advancement devices (MADs) are known to have benefitted [11]. Complete edentulism adds to the worsening of OSA and reduces the number of available therapeutic strategies, with CPAP generally being the treatment of choice. The efficacy of CPAP is said to range from 29 to 83%, which purely relies on patient adherence/non-adherence. Issues with mask discomfort, nasal dryness or congestion, and difficulty adapting to the pressure have been identified as barriers to patient compliance, with many patients rejecting CPAP therapy within the first few months after initiation [3,7]. In completely edentulous patients, the absence of dentition causes a loss of vertical dimension, which leads to morphological changes in the upper airway, a decrease in the retropharyngeal space, and a decrease in the size and tone of the pharyngeal musculature [5,8]. MAD has shown a significant reduction in the apnea-hypopnea index (AHI). A total increase in the volume of the upper airway was seen, predominantly by increasing the volume of the velopharynx, which led to changes in the surrounding soft tissue and bony structures [12]. However, narrowing of the posterior airway space is seen due to encroachment of tongue space by the prosthesis, and thus, fabrication of MAD for an edentulous patient is a challenging task [9].

## Case presentation

A 75-year-old male patient reported to the Government Medical College and Hospital, Kota, Rajasthan, India, with the complaint of excessive daytime sleepiness and snoring. All the necessary checkups were done, and the patient was diagnosed with mild obstructive sleep apnea. The sleep analysis report recorded an apnea-hypopnea index (AHI) of 7.3. Completely edentulous arches with an enlarged tongue are seen due to the long edentulous period. Medical diagnosis by home sleep test (HST). The diagnosis of mild sleep apnea is supported based on the report. Apnea-hypopnea index (AHI) of 7.3, supine AHI: 7.4, and non-supine AHI: 0.0.

The following oximetry findings suggest possible hypoxemia. The oxygen saturation was <86% for more than 99 minutes. The minimum oxygen saturation recorded was at or below 62%.

Assessment can also be done by lateral cephalogram with the appliance in position to check the anterior and posterior pharyngeal spaces. There the patient was advised to CPAP therapy and was also informed about oral appliance therapy.

Later, the patient reported to the Department of Prosthodontics, Daswani Dental College, Kota, Rajasthan, India. The patient had completely edentulous maxillary and mandibular arches and was a complete denture wearer for six to seven years. A radiographic examination (orthopantomogram and lateral cephalogram) was done. Clinical examination revealed an enlarged tongue size and excessive fatty musculature in the cheeks and around the neck. A mandibular advancement device was planned to be fabricated to bring the mandible forward, which in course will lead to an increase in posterior pharyngeal space.

Maxillary and mandibular preliminary impressions were made using a modeling plastic impression compound (Y-dents impression compound; MDM Corp., Delhi, India). Final impressions were made using light-body rubber base impression material (Photosil light body, DPI, Mumbai, India), in border-molded (Green stick compound, DPI, Mumbai, India) custom trays (Cold cure acrylic repair material, DPI RR, Mumbai, India). Care was taken to attain maximum functional extension of the lingual borders. The definitive casts were duplicated using putty of additional silicone material (Photosil, DPI, Mumbai, India). On the duplicated casts, wax (Modelling wax, DPI, Mumbai, India) occlusal rims were then fabricated.

**Figure 1:**
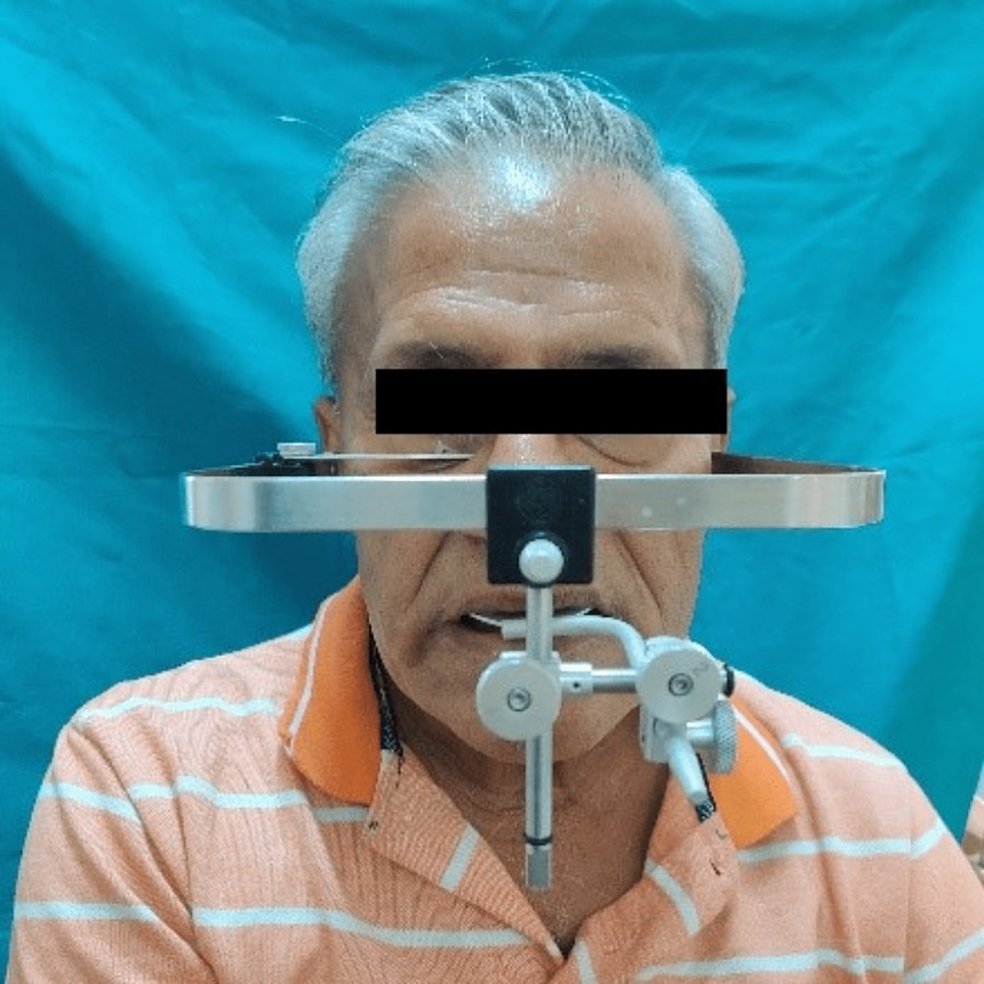
Face bow transfer.

With the patient’s existing vertical dimension of occlusion, the maxillomandibular relation was recorded. The face bow transfer was made with the wax wafer technique (Figure 1), and the maxillary cast was mounted on the Hanau articulator using the indirect method.

**Figure 2:**
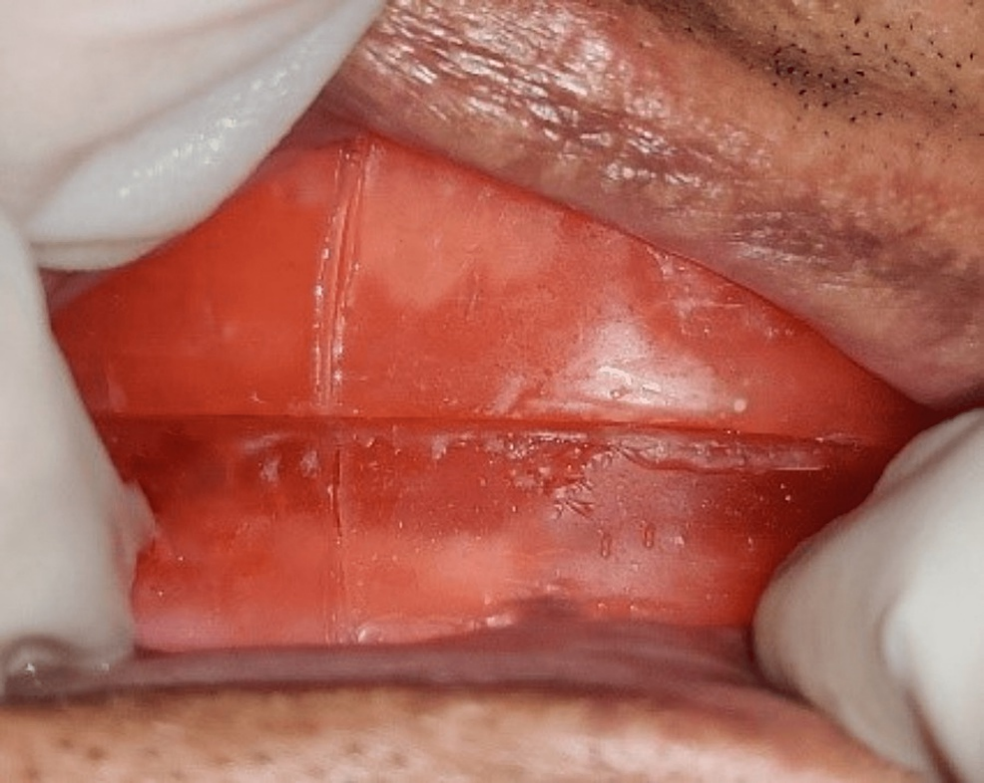
Centric relation.

**Figure 3:**
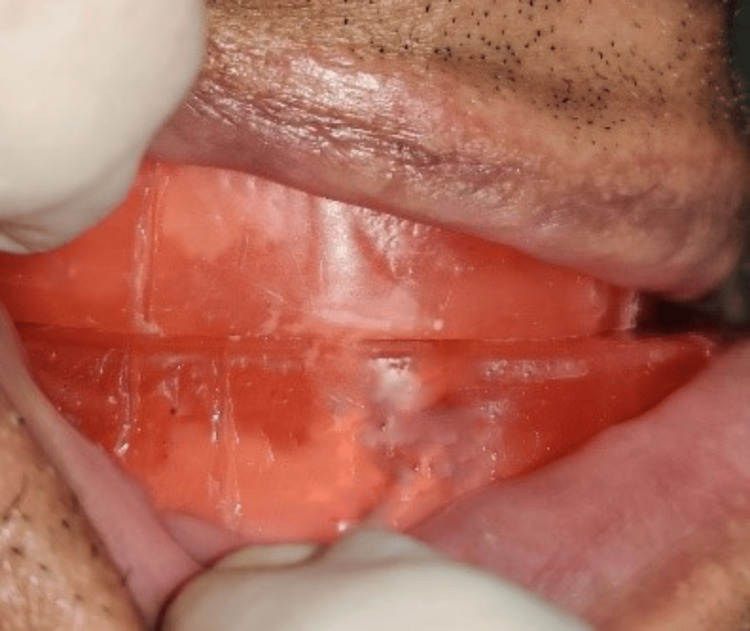
Maximum protruded position of the mandible.

**Figure 4:**
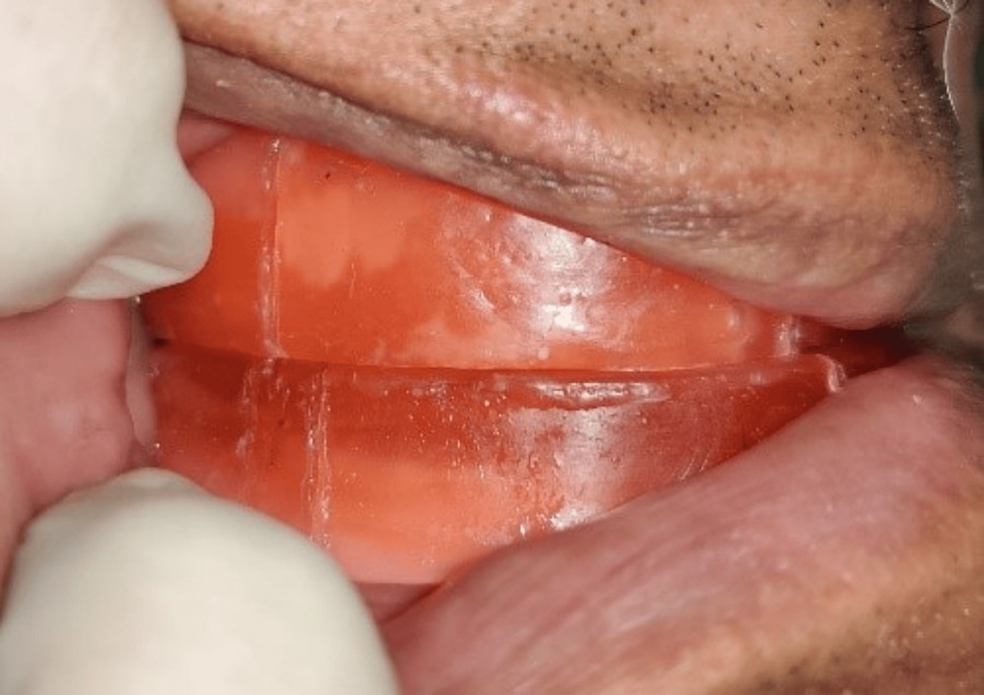
75% of the maximum protruded position of the mandible.

Later, at the centric relation position, vertical markings were made on both maxillary and mandibular occlusion rims bilaterally in the premolar region (Figure 2). The patient was then asked to protrude maximally, and at this maximal protrusion position, another line was marked on the mandibular rim corresponding to the centric relation line in the maxillary rim (Figure 3). The distance between the two marks was ascertained, and then 75% of the distance from the centric relation line was marked. The mandibular rim was then made to occlude so that the centric relation line of the maxillary rim coincided with the 75% line (drawn as previously mentioned) in the mandibular occlusion rim (Figure 4). The maxillomandibular relation was recorded at that position with occlusal registration paste (zinc oxide eugenol (ZNOE)), and the casts were articulated.

**Figure 5:**
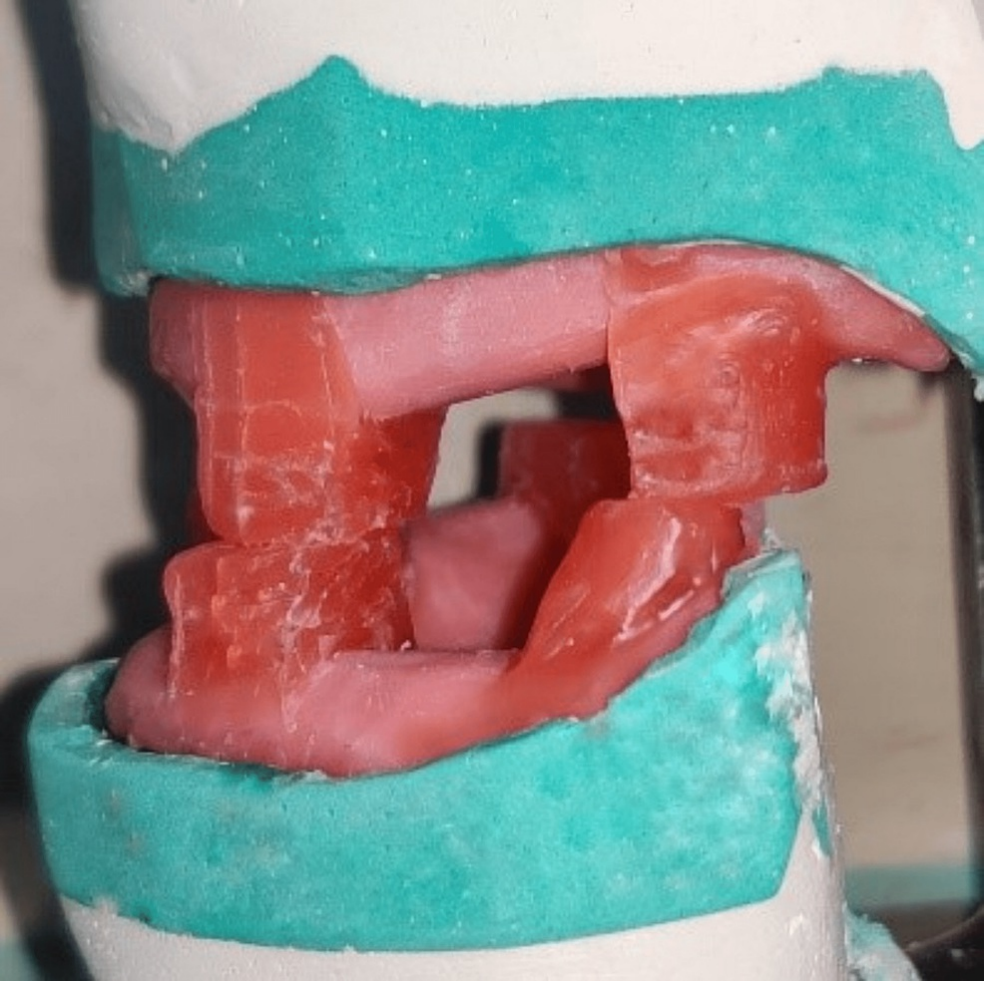
Wax-up of upper and lower members.

Wax-up was done, and anterior and posterior pillars were made with 2-3 mm of spacing between both rims. Anterior pillars are in the premolar region, and in the posterior region, the retromolar pad touches the upper posterior pillar. Also, an anterior open space was given to assist with ventilation (Figure 5). The lingual surface of the pillars was made concave to provide free space for the enlarged tongue.

**Figure 6:**
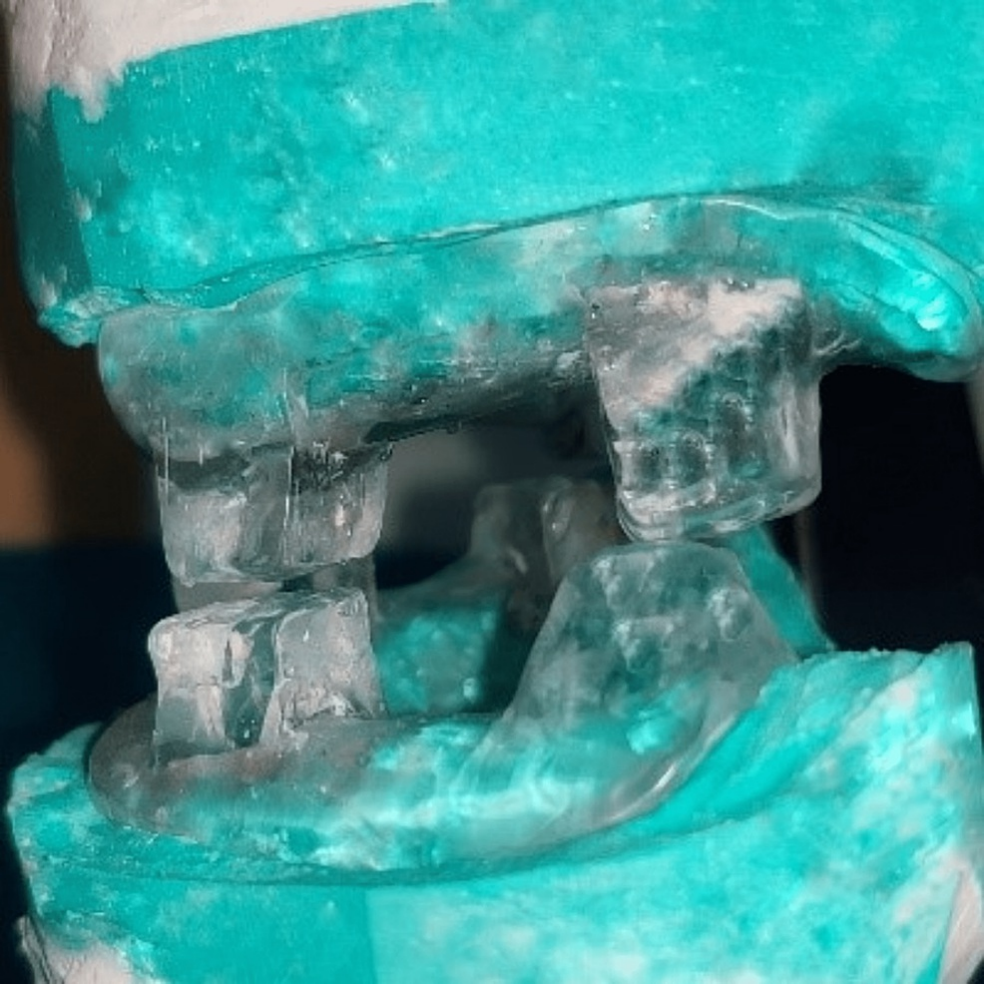
Acrylized upper and lower members with 2-3 mm space between them.

**Figure 7:**
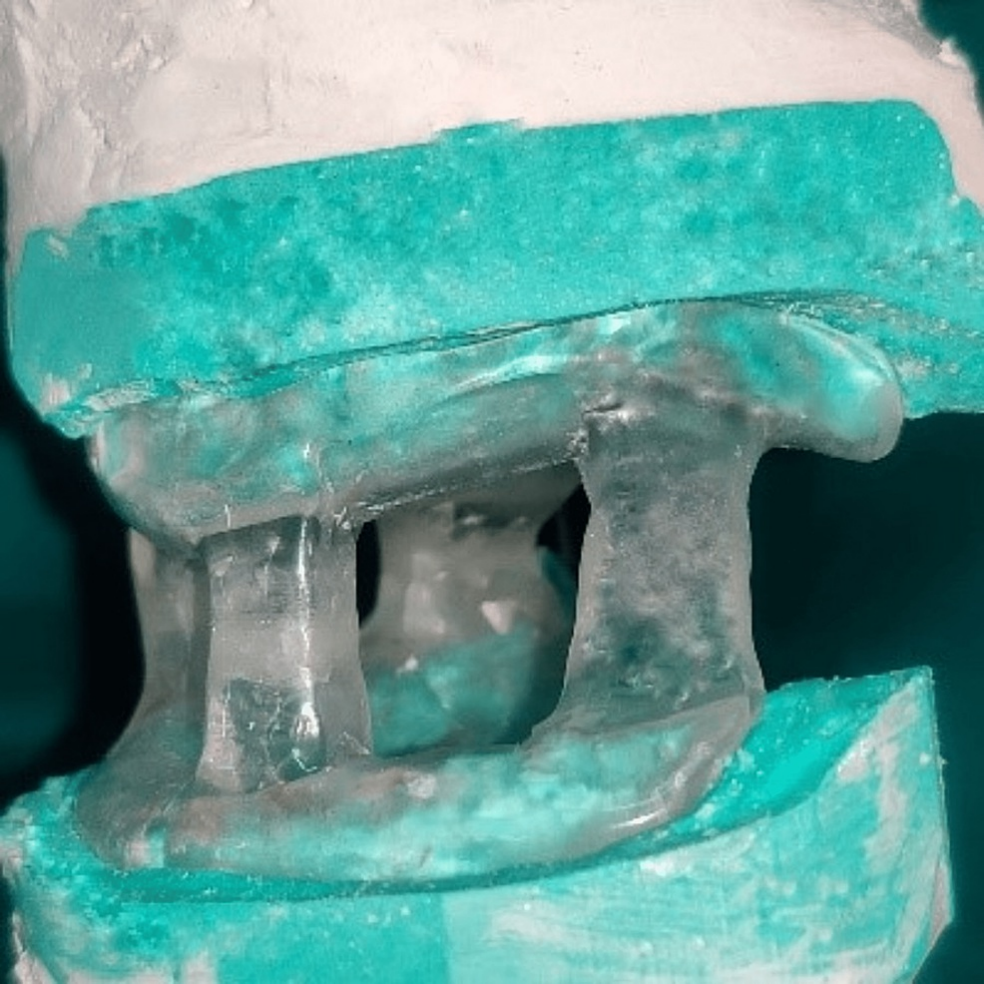
The single unit MAD appliance. MAD: mandibular advancement device.

The upper and lower wax-up were placed on the original final casts to ensure exact retention and stability of bases. They were acrylized separately with a heat-cured clear acrylic resin material (Heat-cured clear denture base material, DPI, Mumbai, India). The acrylized upper and lower members were placed on the mounted articulator (Figure 6). The space that was given between upper and lower pillars were later filled with autopolymerizing acrylic resin material (self-cure clear acrylic material). It was made like a single-unit appliance (Figure 7).

**Figure 8:**
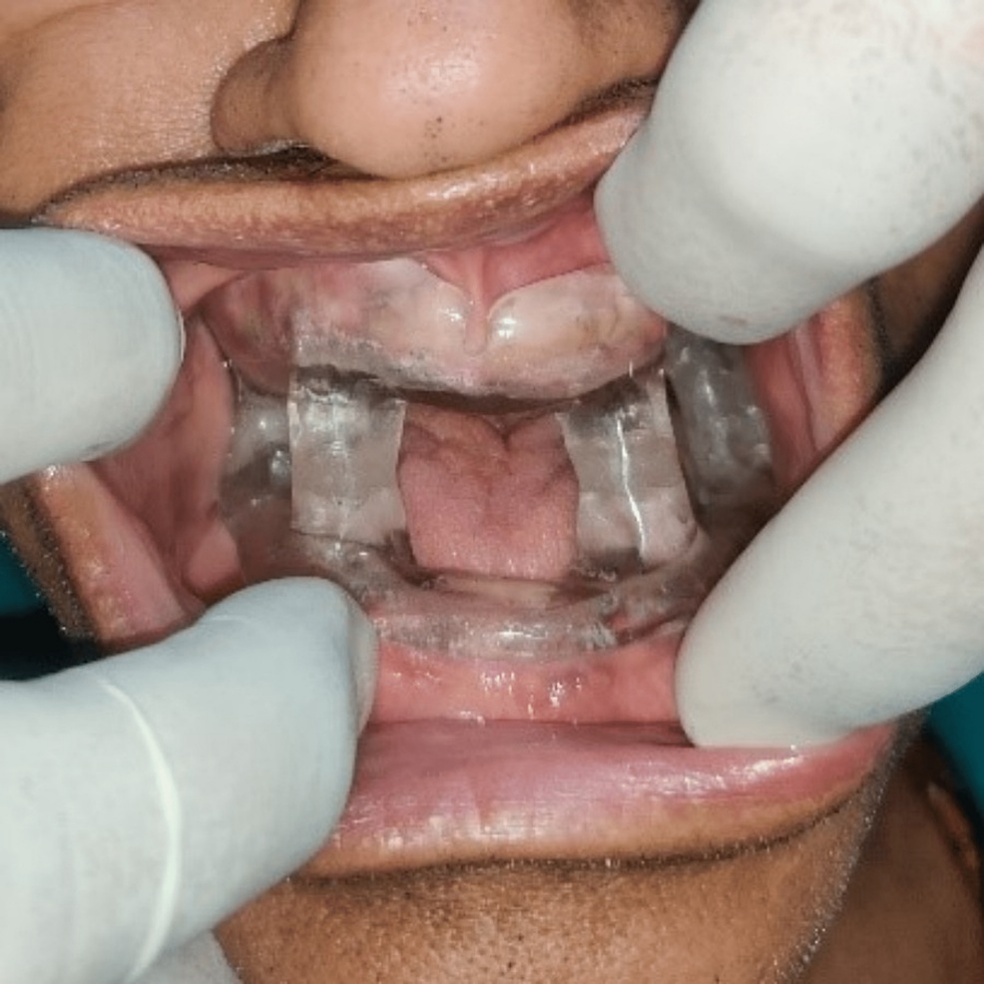
Intra-oral view of the appliance.

**Figure 9:**
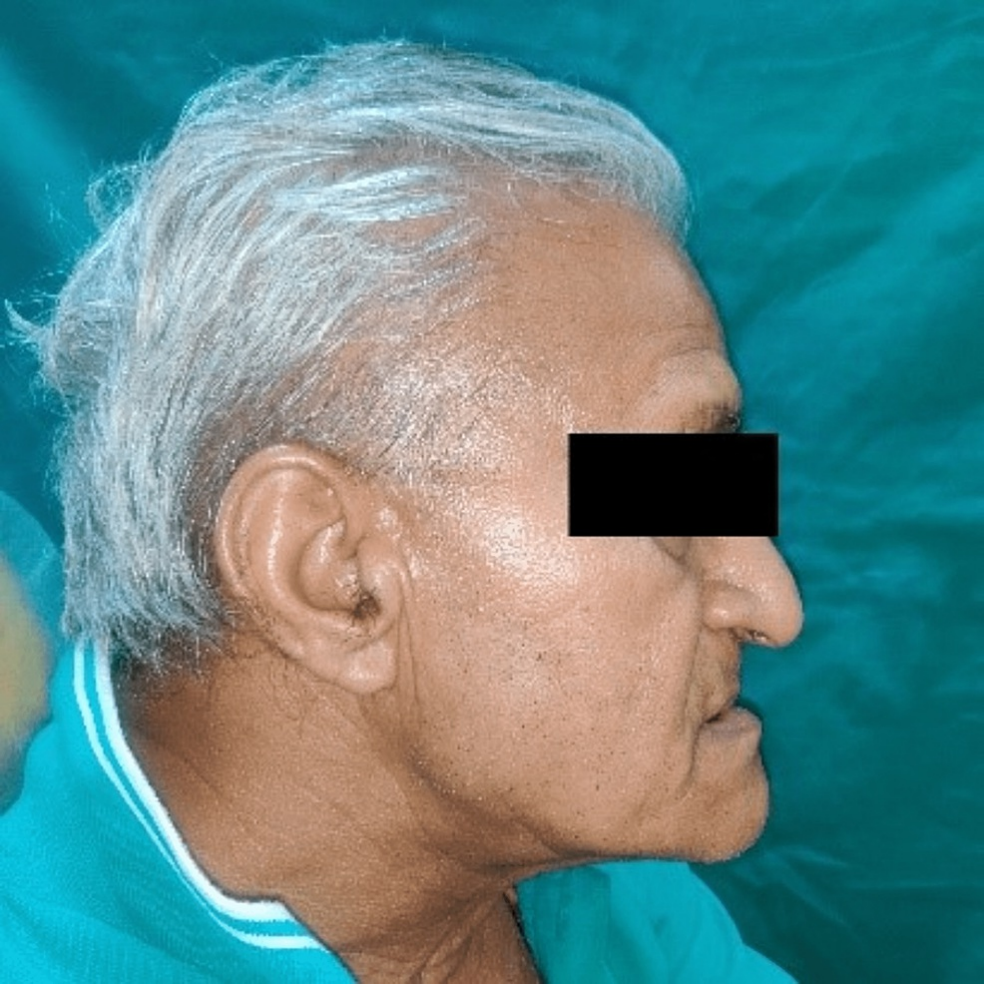
Lateral profile view.

Use pressure-indicating paste clinically to reveal possible sources of tissue irritation and adjust the positions as needed. The intra-oral view of the appliance is shown in Figure 8. A lateral profile view of the patient wearing the MAD appliance shows protruded mandible (Figure 9).

Instruct the patient on how to insert and remove the prosthesis. Provide counseling for the care and cleanliness of the prosthesis at home. Complete dentures should be removed for a specific period during the day to avoid or minimize the adverse effects of continuous wear. At the follow-up, evaluate and assess the patient’s sleep and check for the onset of hypersomnolence and other related symptoms. It should have good stability and retention so that the patient can wear it comfortably throughout the night and not cause myofascial or temporomandibular joint (TMJ) symptoms. If discomfort develops, the prosthesis could be separated and the necessary changes made.

Post-insertion instructions were provided at the time of MAD's insertion. The patient was advised to wear the appliance for one to two hours during the daytime for the first week to make him accustomed to the appliance. The patient was recalled after a week to check for the progress made and was later advised to start wearing the appliance during the night as well. At the first monthly evaluation, the patient’s daytime sleepiness was reduced and his sleep improved at night.

## Discussion

Most prevalent minor and temporary side effects instigated with MAD are occlusal changes, mucosal dryness or hypersalivation, transient tooth or TMJ pain, and masticatory muscle or myofascial discomfort in the morning [4].

There were only a few methods for fabrication of MAD in edentulous cases stated in history as in reviewed many articles. In the year 1990, the first such treatment for sleep apnea in an edentulous patient was done by Meyer et al. An appliance with 5-8 mm of protrusive position was made at an increased vertical dimension [13].

In a study conducted to see the effects of an oral appliance with different mandibular protrusive positions (i.e., at 0%, 25%, and 75%) and at a constant vertical dimension, AHI values were recorded to be lower in the 50% and 75% positions [14].

Keyf et al., fabricated an appliance for the edentulous arches in which an interocclusal wafer was screwed between the maxillary and mandibular dentures, making this a single unit. The interarch distance between the dentures was increased with the placement of the wafer, which made the MAD appliance self-retentive even at rest. Since this appliance was a modified form of dentures that were already in use, no discomfort or fresh complaints of soreness or ulceration were reported.

To avoid any possible anterior impingement of the glenoid fossae by the condyles, the MAD was made at 70% of the maximum mandibular protrusion [15]. The long-term negative side effects produced by excessive advancement procedures on occlusion and temporomandibular joints are still unknown. Johal and Battagel found that the space between the base of the tongue and the posterior pharyngeal wall decreased when there was an increase in the occlusal vertical dimension [16].

The principle aim in the fabrication of this appliance was to increase the space between the base of the tongue and the posterior pharyngeal wall. This was attained by the protrusion of the mandible without increasing the vertical dimension of occlusion [10].

## Conclusions

With the fabrication of MAD in this completely edentulous patient, a consequential increase in both anterior and posterior pharyngeal space was seen. This revealed a definite improvement in the AHI index. Also, the ease of fabrication of this particular appliance is foreground to other methods.
